# Supplementary figures and images for: Genetic Diversity and Selection Signatures of Lvliang Black Goat Using Genome-Wide SNP Data
Source: Animals (Basel). 2024 Nov 3;14(21):3154. doi: 10.3390/ani14213154 (PMC11544794; doi:10.3390/ani14213154)

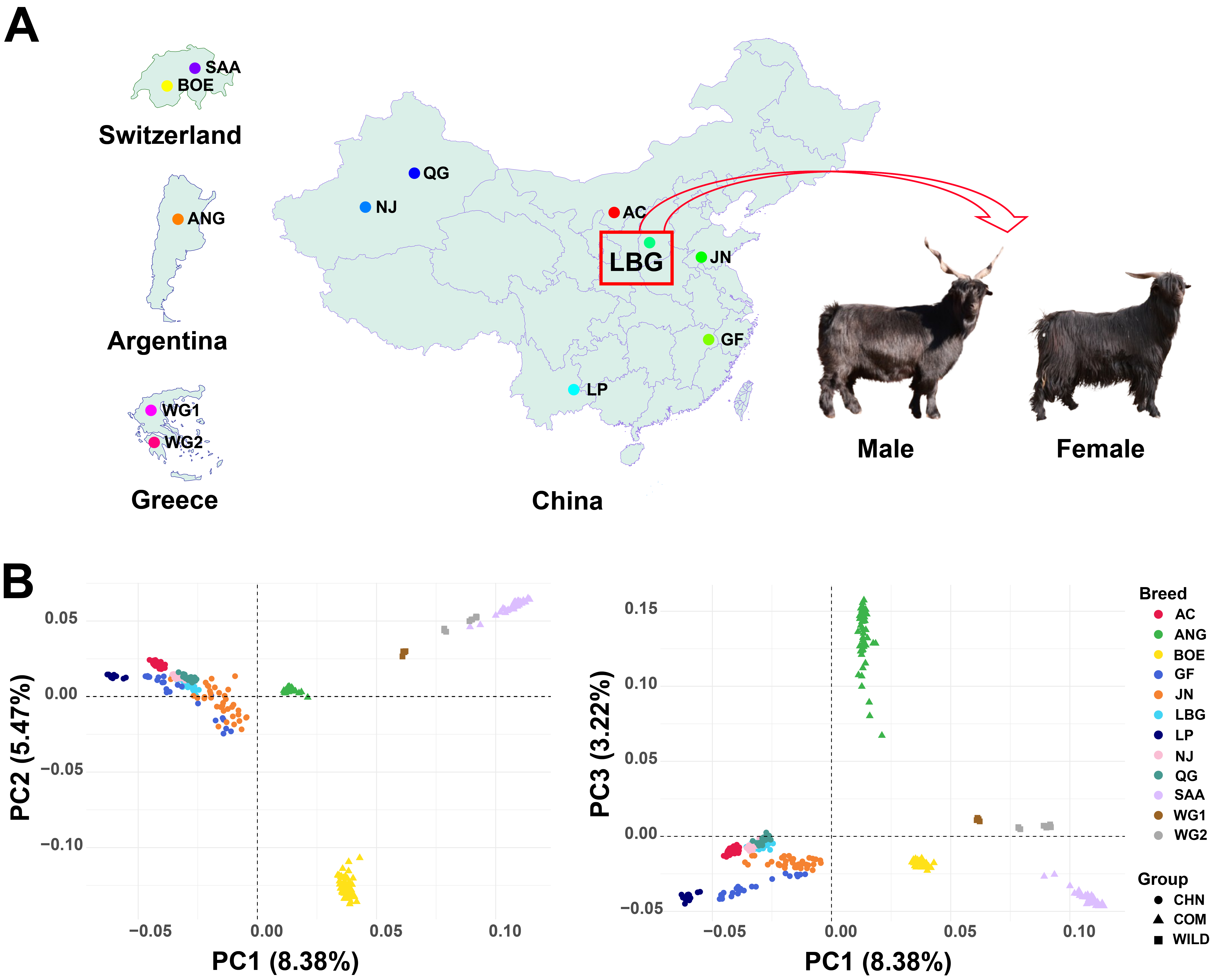

Supplement: Supplementary file 1 [file animals-14-03154-s001.zip › figure3.png]

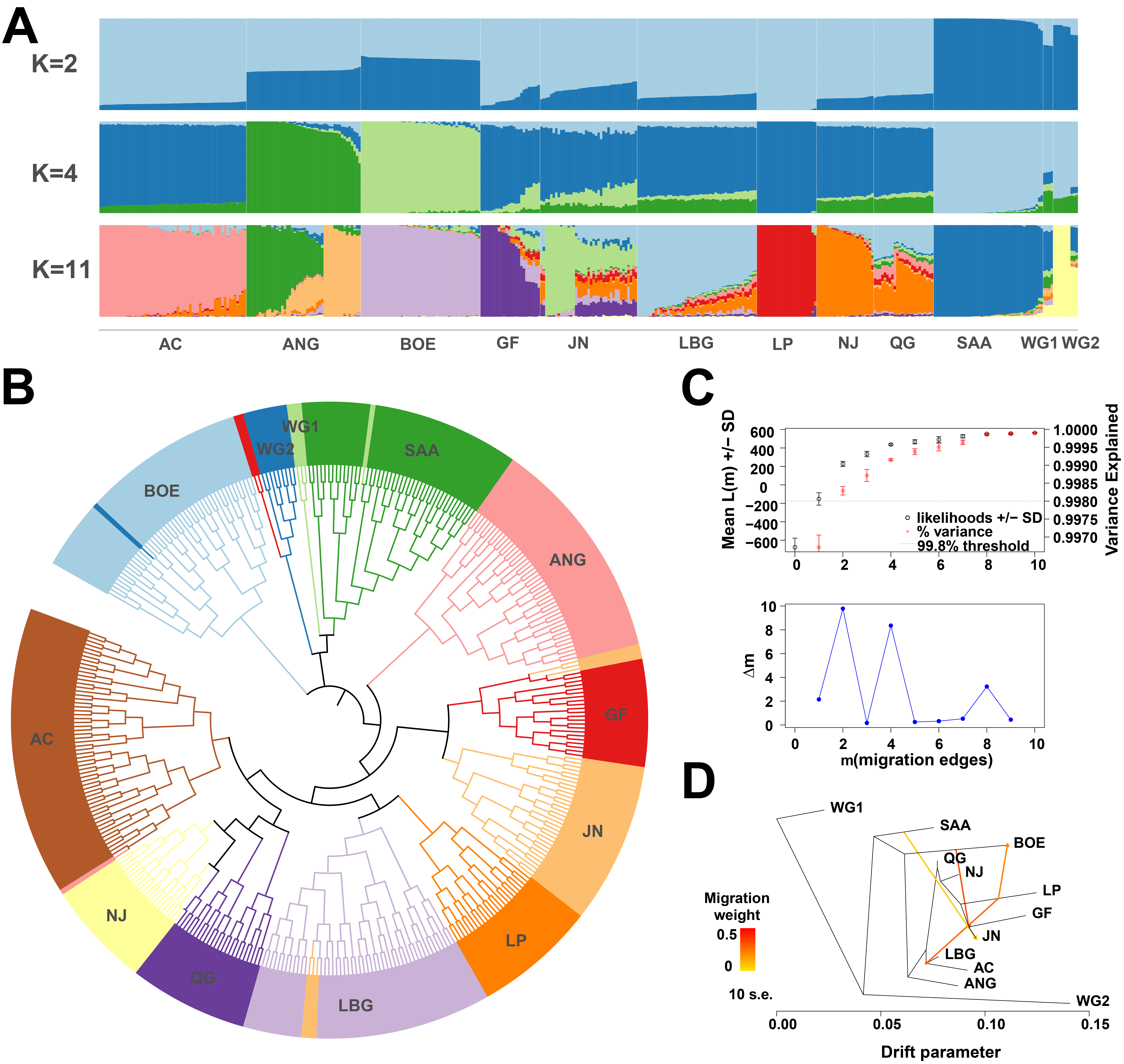

Supplement: Supplementary file 1 [file animals-14-03154-s001.zip › figure4.png]

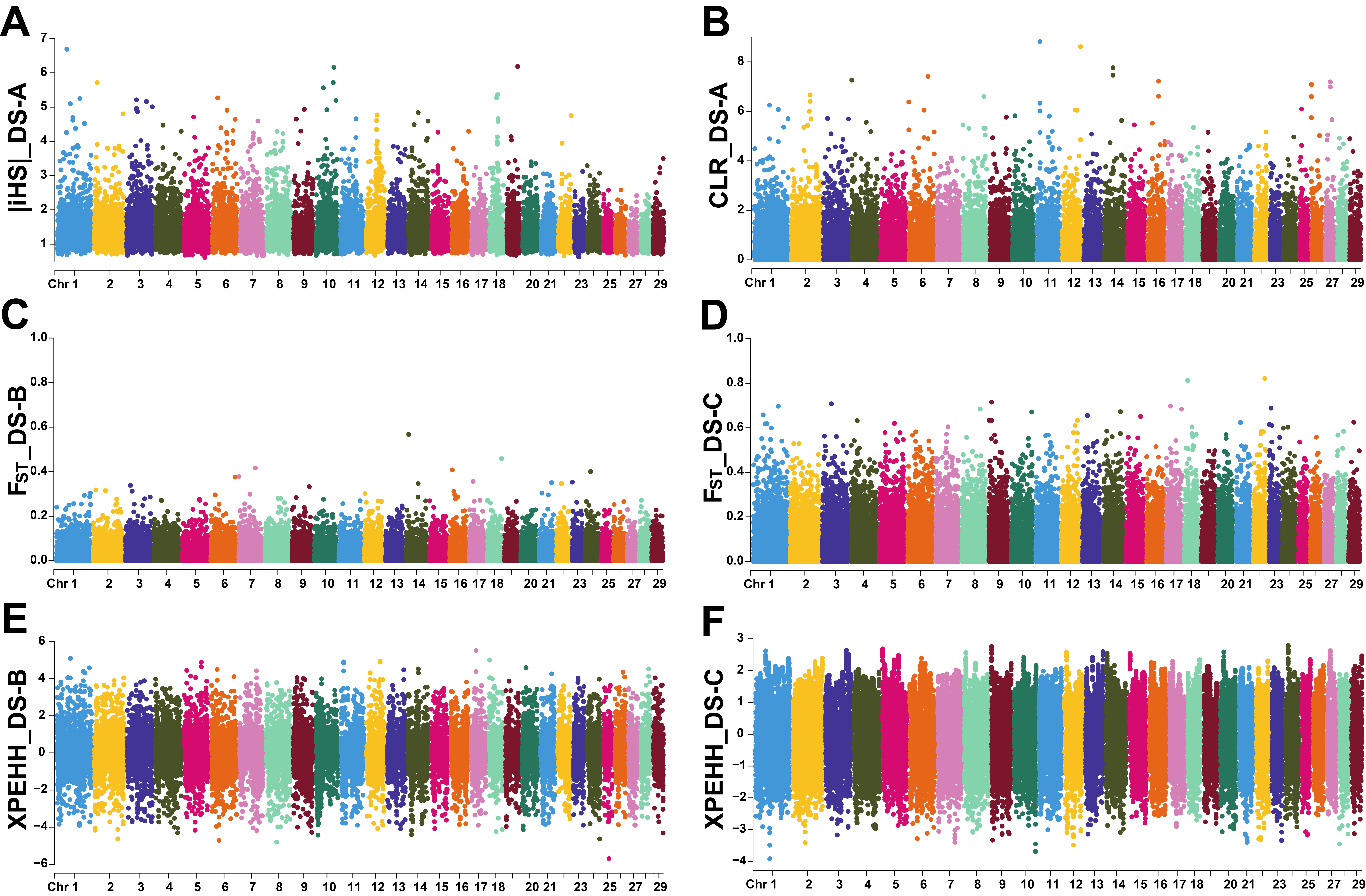

Supplement: Supplementary file 1 [file animals-14-03154-s001.zip › figure5.png]

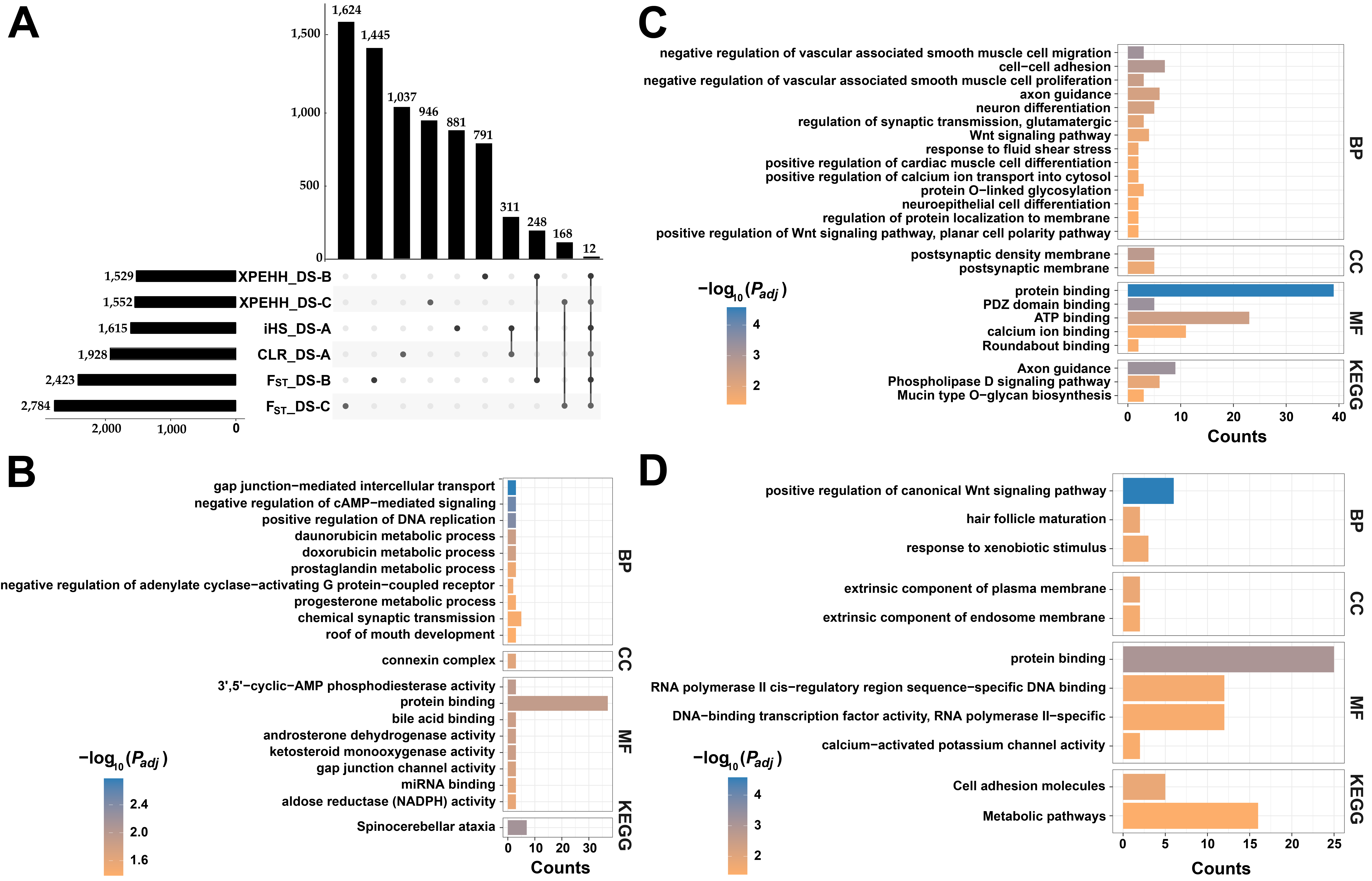

Supplement: Supplementary file 1 [file animals-14-03154-s001.zip › figure6.png]

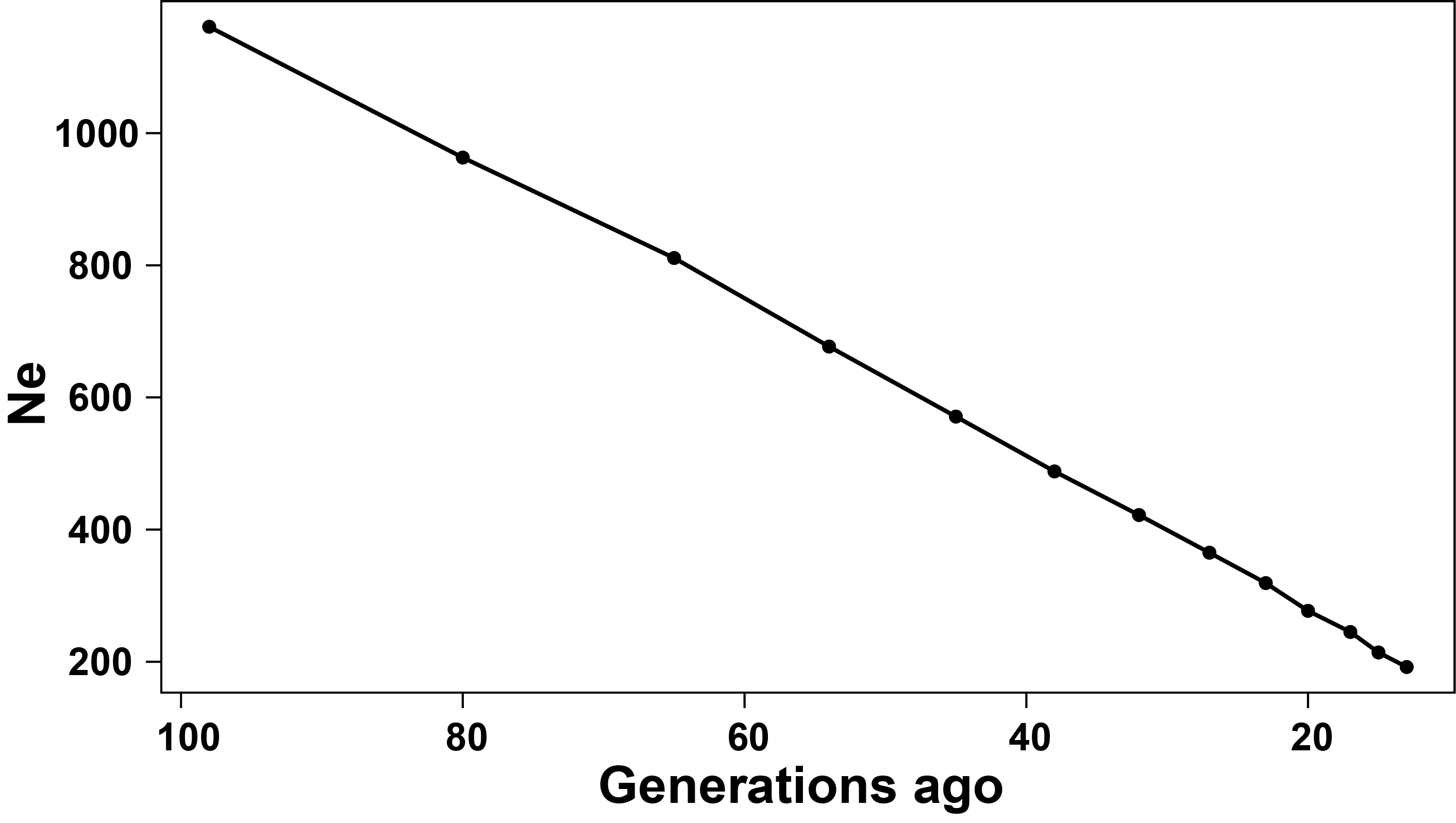

Supplement: Supplementary file 1 [file animals-14-03154-s001.zip › FigureS1.png]

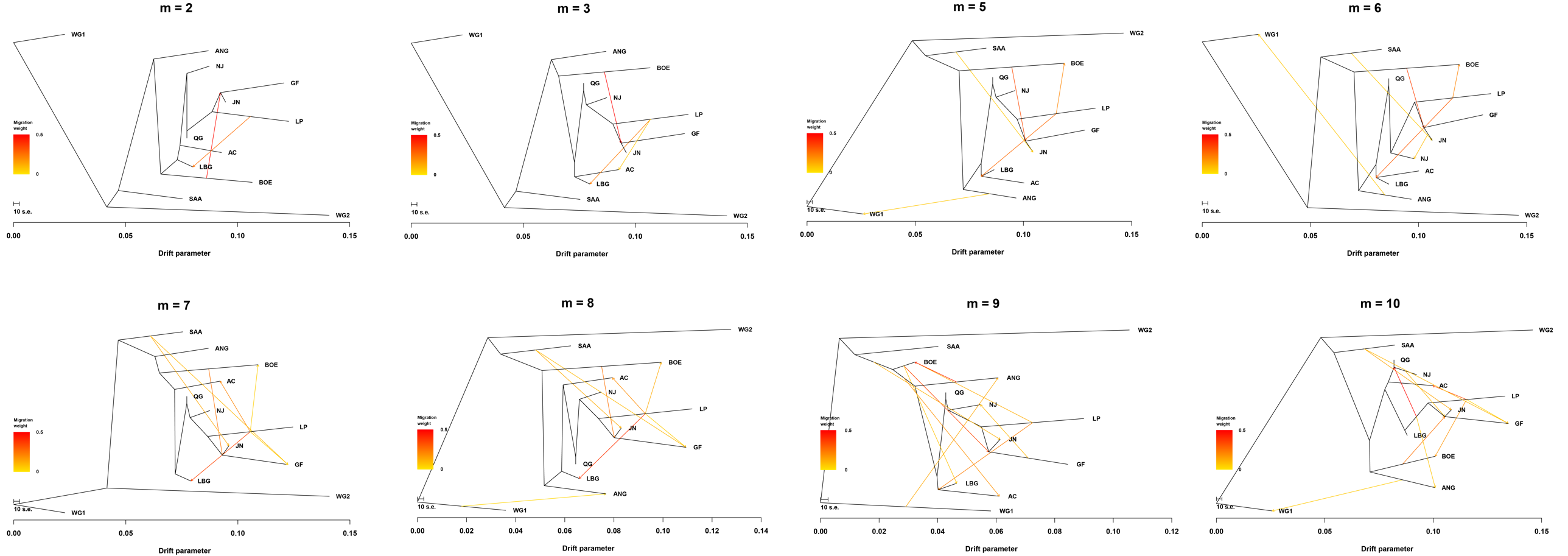

Supplement: Supplementary file 1 [file animals-14-03154-s001.zip › FigureS2.png]

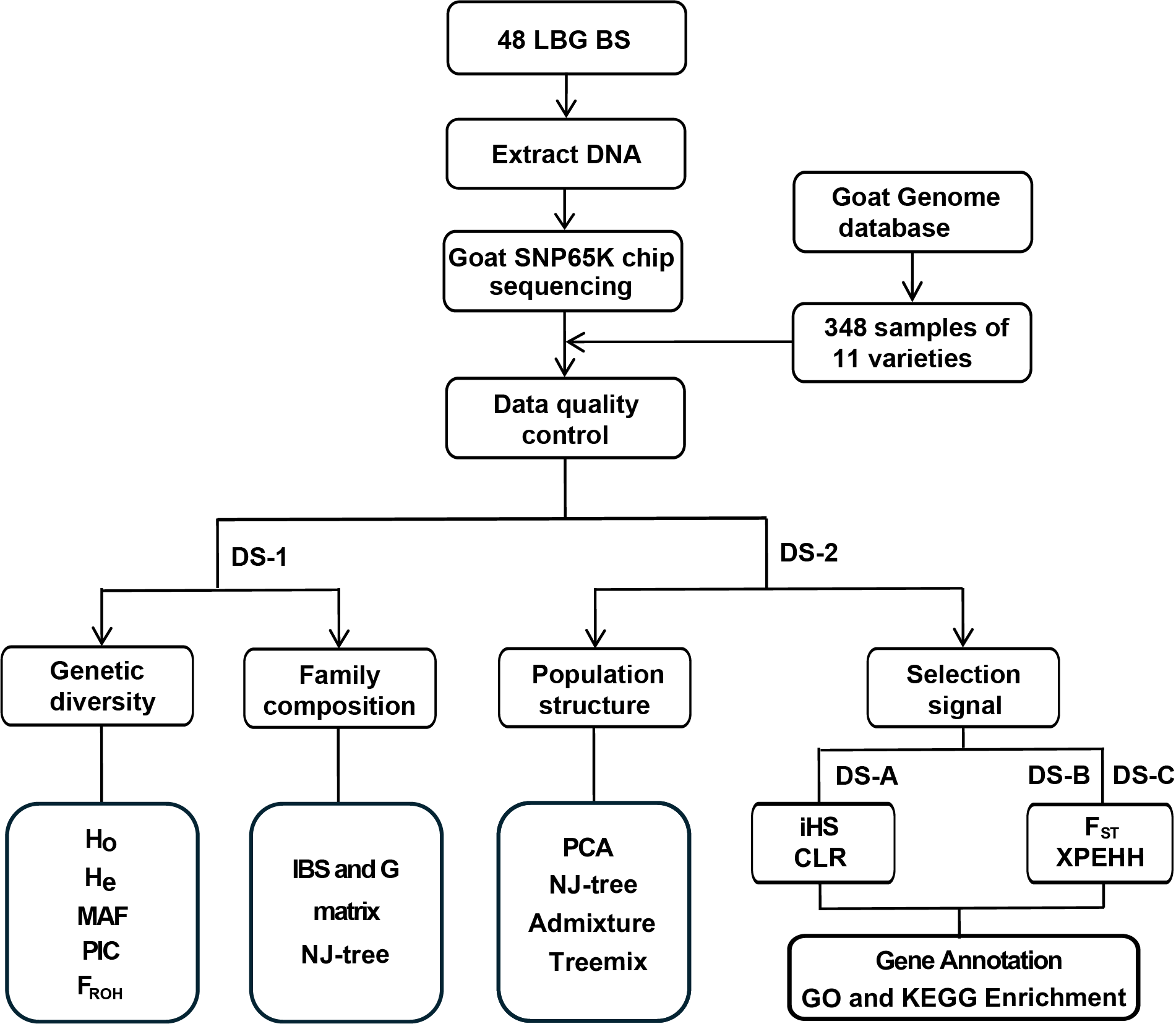

Supplement: Supplementary file 1 [file animals-14-03154-s001.zip › figure1.png]

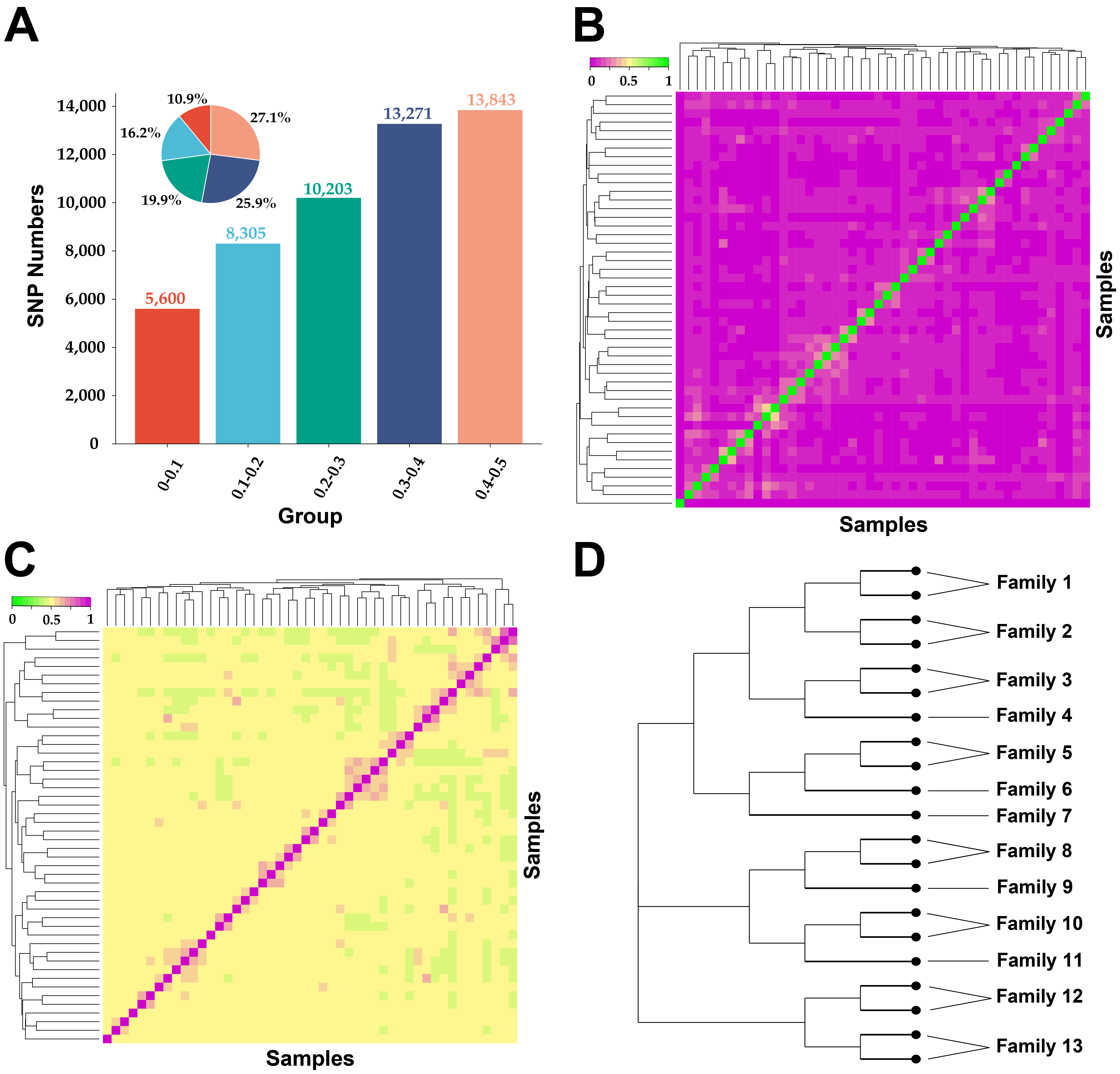

Supplement: Supplementary file 1 [file animals-14-03154-s001.zip › figure2.png]
